# Retraction: Physical fitness characteristics and comprehensive physical fitness evaluation model of basketball players based on association rule algorithm

**DOI:** 10.1371/journal.pone.0351202

**Published:** 2026-06-23

**Authors:** The PLOS One Editors

The *PLOS One* Editors retract this article [1] due to the following concerns:

- Non-compliance with the PLOS Human Research Subjects policy as the article describes the collection of human participant data, but provides no information on ethics approval or consent.
- Non-compliance with the PLOS Data Availability policy as the underlying data were not provided with the article, contrary to the Data Availability statement.
- Data collection methods, the type of data collected, and the sample population are not described in sufficient detail to reproduce the study or to support the findings and conclusions of the study.
- The articles cited as References 2 and 3 were retracted prior to the publication of [1] and appear to be irrelevant in the cited context.
- Several statements in the introduction are not supported by references, and several references, including References 6, 7, and 19, appear to be irrelevant in the cited context.
- There are multiple inconsistencies in the article; for example, the Proposed methodology section refers to “a database of sales transactions” and Figs 8 and 9 are described as presenting results about transactions, despite the study being about basketball players.

We regret that the issues were not identified prior to the article’s publication.

The author did not agree with the retraction.
